# TRPM8 and RAAS-mediated hypertension is critical for cold-induced immunosuppression in mice

**DOI:** 10.18632/oncotarget.24356

**Published:** 2018-01-30

**Authors:** Hao Chan, Hsuan-Shun Huang, Der-Shan Sun, Chung-Jen Lee, Te-Sheng Lien, Hsin-Hou Chang

**Affiliations:** ^1^ Institute of Medical Sciences, Tzu-Chi University, Hualien, Taiwan; ^2^ Center for Prevention and Therapy of Gynecological Cancers, Department of Research, Buddhist Tzu Chi General Hospital, Hualien, Taiwan; ^3^ Department of Molecular Biology and Human Genetics, Tzu-Chi University, Hualien, Taiwan; ^4^ Department of Nursing, Tzu Chi College of Technology, Hualien, Taiwan

**Keywords:** cold exposure, TRPM8, immunosuppression, renin–angiotensin–aldosterone system, hypertension

## Abstract

Mechanisms underlying cold-induced immunosuppression remain unclear. Here we found that cold exposure leads to transient receptor potential melastatin 8 (TRPM8)-dependent, renin–angiotensin–aldosterone system (RAAS)-mediated hypertension, which subsequently induces small molecule and fluid extravasation, increases plasma Ig levels, and elicits immunosuppression. An effect is similar to the clinically-used immunosuppressive treatments of intravenous immunoglobulin (IVIg) against various inflammatory diseases, such as immune thrombocytopenia (ITP). Essential roles of TRPM8 and Ig in cold-induced immunosuppression are supported by the cold-mediated amelioration of ITP and the cold-mediated suppression of bacterial clearance, which were observed in wild-type mice but not in Ig- and TRPM8-deficient mutants. Treatment with antihypertensive drugs aliskiren and losartan drastically reversed high plasma Ig levels and ameliorated cold-induced immunosuppression, indicating the involvement of the RAAS and hypertension. These results indicated that the natively increased plasma Ig level is associated with immunosuppression during periods of cold exposure, and antihypertensive drugs can be useful to manage cold-induced immunosuppression.

## INTRODUCTION

The ancient physicians Hippocrates (460–370 BC) and Zhang Zhongjing (150–219 AD) have documented the relationship between diseases and cold exposure [1, 2]. Many recent studies have reported that respiratory tract infections, pneumonia, and the outbreak of influenza viruses are associated with seasonal reductions in temperature [3–6]. Despite hundreds of years of study, the mechanisms underlying cold exposure-associated infections and the related immune system disturbances remain elusive; millions of people are still affected by seasonal infections every year [7]. Various hypotheses have been postulated to explain cold exposure-induced immunosuppression. Studies have suggested that cold exposure can result in the production of immunosuppressive corticosteroids through the hypothalamic–pituitary–adrenal (HPA) axis [8, 9]. Another study suggested that a drop in the temperature reduces local circulation and restricts the functioning of leukocytes in the upper airway tract [10]. However, the detailed mechanism and its effect on immunosuppression require additional investigation.

Intravenous immunoglobulin (IVIg) consists of pooled immunoglobulin (Ig) fractions purified from thousands of donor plasma samples. Although rescue mechanisms of IVIg on autoimmune and inflammatory diseases, such as immune thrombocytopenia (ITP) and Kawasaki disease, remain unclear, administering a high dose of IVIg to increase circulating Ig levels is an effective treatment for these diseases [11]. As IVIg is an artificial treatment, these evidences let us wondered whether there exists a native physiological response by upregulating the circulating Ig levels to suppress the immune system. Cold exposure-induced immunosuppression has caught our attention, because cold exposure is known to elicit hypertension [12], hypertension is associated with induction of high circulating Ig levels [13], and high circulating Ig levels (e.g. IVIg treatments) is able to suppress the immune response [11]. To formulate a more feasible approach, using clues that have not been previously linked together, we hypothesize that cold exposure increases circulating Ig levels through following processes. To prevent heat loss, cold exposure induces vascular constriction and hypertension [14–16]; these responses can lead to fluid extravasation [17]. Because the vascular wall is a semipermeable barrier that allows the penetration of small molecules, fluid extravasation should theoretically result in retention and increased levels of large plasma protein molecules, such as Ig, within blood vessels. This could be why some patients with hypertension [13, 18, 19] or those persons exposed to a cold environment [20] exhibit a convergent phenotype with increased plasma Ig levels in these different conditions. On the basis of these findings, we hypothesize that cold-induced immunosuppression is a native IVIg-like behavior mediated by a high plasma Ig level in response to cold exposure. If this hypothesis is correct, we may be able to overcome cold-induced immunosuppression by managing cold exposure–induced hypertension.

In this study, we used the following two assay systems to analyze IVIg- and cold exposure-mediated immunosuppression: amelioration of ITP and reduced clearance of bacteria. P-selectin mutant mice were used because they have a defective phenotype in response to IVIg-induced anti-inflammation compared with normal mice [21]. B cell-deficient (BCD) mice (Ig heavy constant mu [*Ighm*]^−*/*−^), which lack the expression of circulating Ig, were used to examine the essential role of circulating Ig in cold-induced immunosuppression. Transient receptor potential melastatin 8 (TRPM8) is a cold receptor primarily expressed on sensory neurons to sense a drop in the temperature [22]. *Trpm8^−/−^* mutant mice were used to investigate whether the TRPM8 pathway is involved in cold-mediated immunosuppression. The relevant mechanisms and potential treatments are discussed.

## RESULTS

### Cold exposure rapidly induces hypertension through the renin–angiotensin–aldosterone system in mice

**Figure 1:**
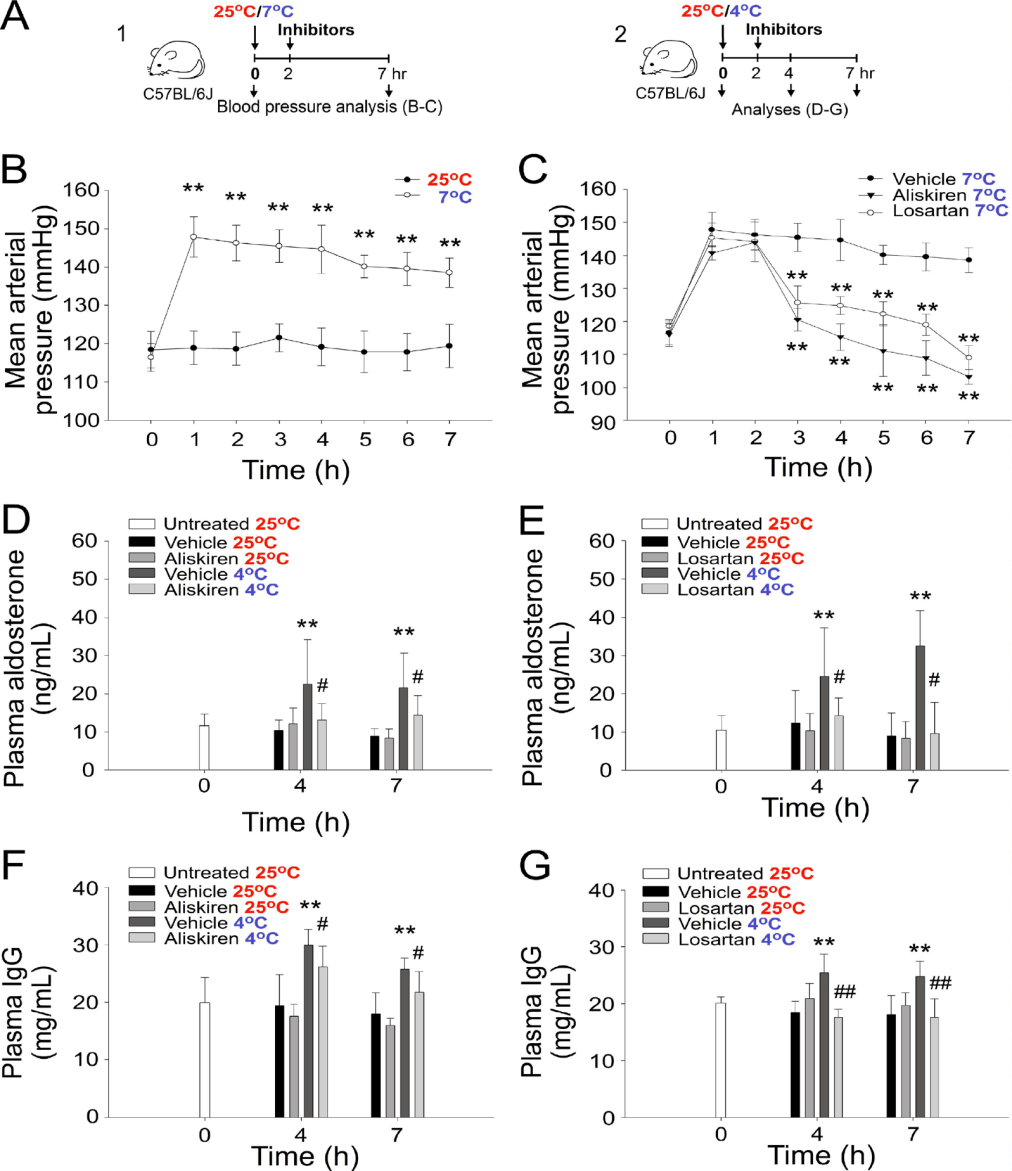
Cold exposure–induced hypertension and increased plasma aldosterone and IgG levels Experiment outlines (**A**). The mean arterial pressure (mmHg) of C57Bl/6J mice exposed to cold environments (7° C) with or without treatment with the antihypertension drugs aliskiren and losartan (**B**, **C**). The plasma aldosterone (**D**, **E**) and IgG (**F**, **G**) levels of C57Bl/6J mice exposed to a 4° C environment with or without treatment with the antihypertension drugs aliskiren (D, F) and losartan (E, G). ^*^P < 0.05 and ^*^P < 0.01 compared with the respective 25° C (B, D, E, F, G) and 7° C (C) groups. All data in this report are presented as the mean ± standard deviation (SD) and are representative of 3–4 independent experiments with two mice per group (*n* = 8 in 0-h groups and *n* = 6 in 1–7-h groups). ^#^*P* < 0.05 and ^##^*P* < 0.01 compared with the respective vehicle group at 4° C (D–G).

Studies have demonstrated that cold exposure for weeks increased blood pressure in mice [16, 23, 24]; however, whether a short-term cold exposure leads to hypertension in hours remains unclear. In the current study, exposure of wild-type mice to a 7° C environment unexpectedly increased the blood pressure of mice within 1 h (Figure 1A experiment outline, 1B, and 1C). In addition, the experimental cold exposure elevated plasma aldosterone levels (Figure 1D and 1E). Inhibitors against renin (aliskiren) and angiotensin (losartan), components of renin–angiotensin–aldosterone system (RAAS), both significantly suppressed cold-induced hypertension and plasma aldosterone levels increased due to cold exposure in mice (Figure 1C–1E). Renin activity analysis also confirmed an effective inhibition of renin by aliskiren (Supplementary Figure 1). Gene expression analysis by quantitative reverse transcription-polymerase chain reaction (qRT-PCR) revealed that RAAS components, such as renin, angiotensin II receptors AT1a, AT1b, AT2 and angiotensin converting enzyme (ACE), all markedly increased after 4 h cold exposure (Supplementary Figure 2A). These results collectively suggest that cold exposure can induce acute RAAS-mediated hypertension within 1 h, and such hypertension is sustainable for hours in mice (Figure 1B and 1C). Notably, plasma IgG levels were positively associated with an increase in aldosterone levels because of cold exposure and could be reversed by aliskiren and losartan treatments during cold exposure (Figure 1F and 1G), suggesting that the RAAS and hypertension modulate plasma Ig levels during cold exposure.

### Cold-induced hypertension causes extravasation of circulating small molecules

**Figure 2:**
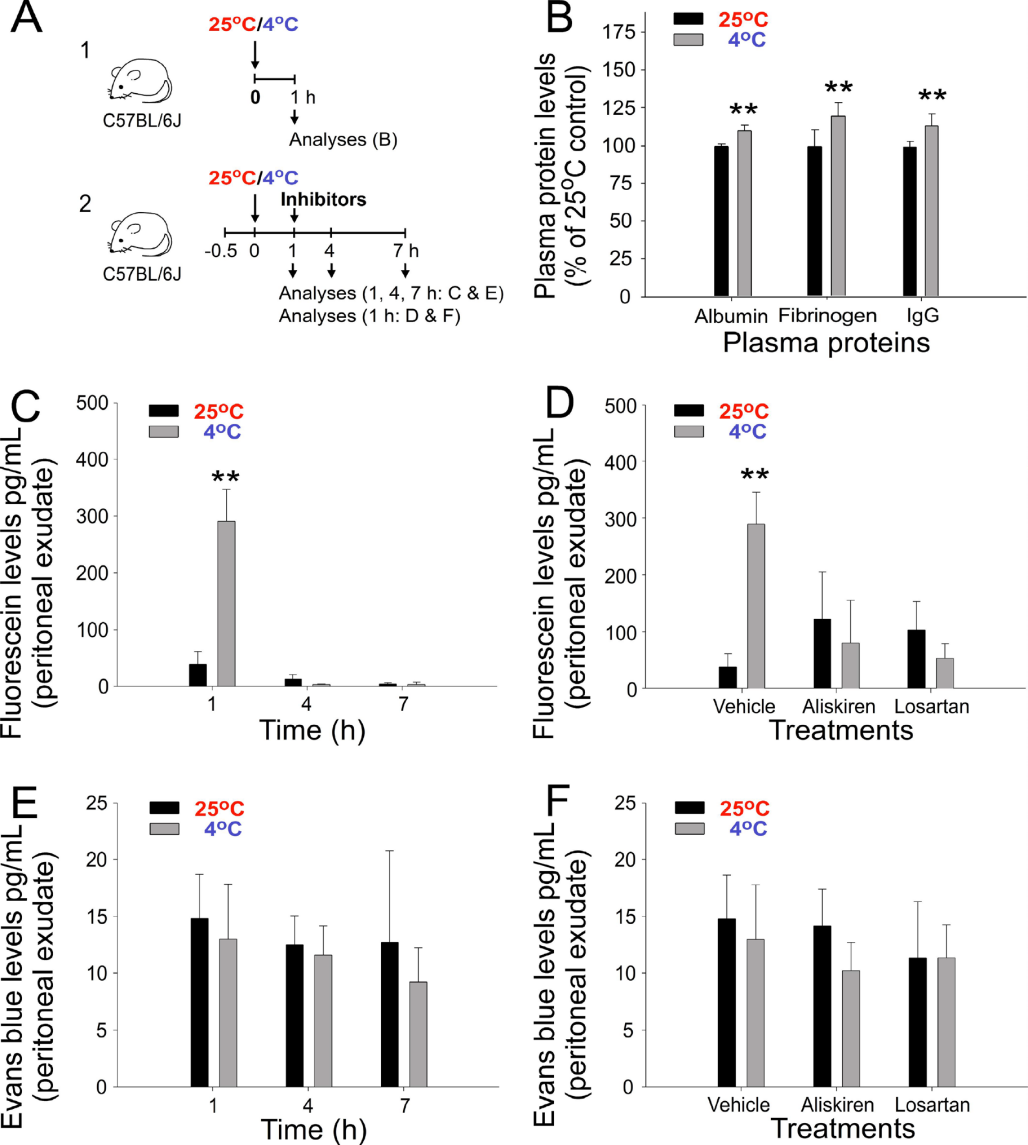
Plasma protein and extravasation of injected fluorescein and Evans blue (EB) dyes after cold exposure in mice Experiment outlines (**A**). Plasma albumin, fibrinogen, and immunoglobulin G (IgG) levels (**B**), and the peritoneal extravasation of fluorescein (**C**, **D**) and EB (**E**, **F**) with or without treatment with aliskiren and losartan (**D**, **F**) is shown. ^*^P < 0.05 and ^*^P < 0.01 compared with the respective 25° C groups. *n* = 10 in D, vehicle groups (five experiments with two mice per group); all other groups, *n* = 6 (three experiments with two mice per group).

To investigate whether in addition to IgG levels, the plasma levels of other proteins are elevated during cold exposure, three major plasma proteins, albumin, fibrinogen, and IgG, were compared and analyzed. The results indicated that the plasma levels of all three tested proteins were elevated after 1 h of cold exposure (Figure 2A experiment outline and 2B), suggesting that this can be a comprehensive regulation, which is not restricted to a particular protein. This finding prompted us to hypothesize that, because the vascular wall is a semipermeable barrier that allows the penetration of small molecules [25], the extravasation of fluids and small molecules during cold exposure [17] should result in retention and increased levels of large plasma protein molecules, such as Ig, within blood vessels. To test this hypothesis, we used the small molecule dyes fluorescein and Evans blue (EB).

In contrast to fluorescein, EB binds to albumin (67,000 daltons) and forms high-molecular-weight dye–protein complexes after intravascular injection [26]. This would theoretically lead to differential extravasation behaviors of fluorescein versus EB. In agreement with our suggestion, an intravascular injection of fluorescein, but not of EB, resulted in a high level of extravasation into the peritoneal cavity during cold exposure (Figure 2C and 2D, 1-h groups and Figure 2E and 2F). Notably, such extravasation of fluorescein can be reversed by treatment with the aforementioned antihypertensive drugs aliskiren and losartan (Figure 2D). In agreement with our hypothesis, these results suggest that the extravasation of small molecules, such as fluorescein, is driven by cold-induced hypertension. The absence of the peritoneal extravasation of EB indicated the retention of circulating albumin and larger molecules, such as Ig, in the mouse blood flow under cold exposure. Because fluid extravasation has been associated with hypertension [17] and the molecular weight of fluorescein (C_20_H_12_O_5_; 332.3 g mol^−1^) is higher than that of water (H_2_O; 18 g mol^−1^), these results collectively suggest that cold exposure induces the extravasation of fluids and small molecules in plasma.

### Cold exposure induces immunosuppression in mice

**Figure 3:**
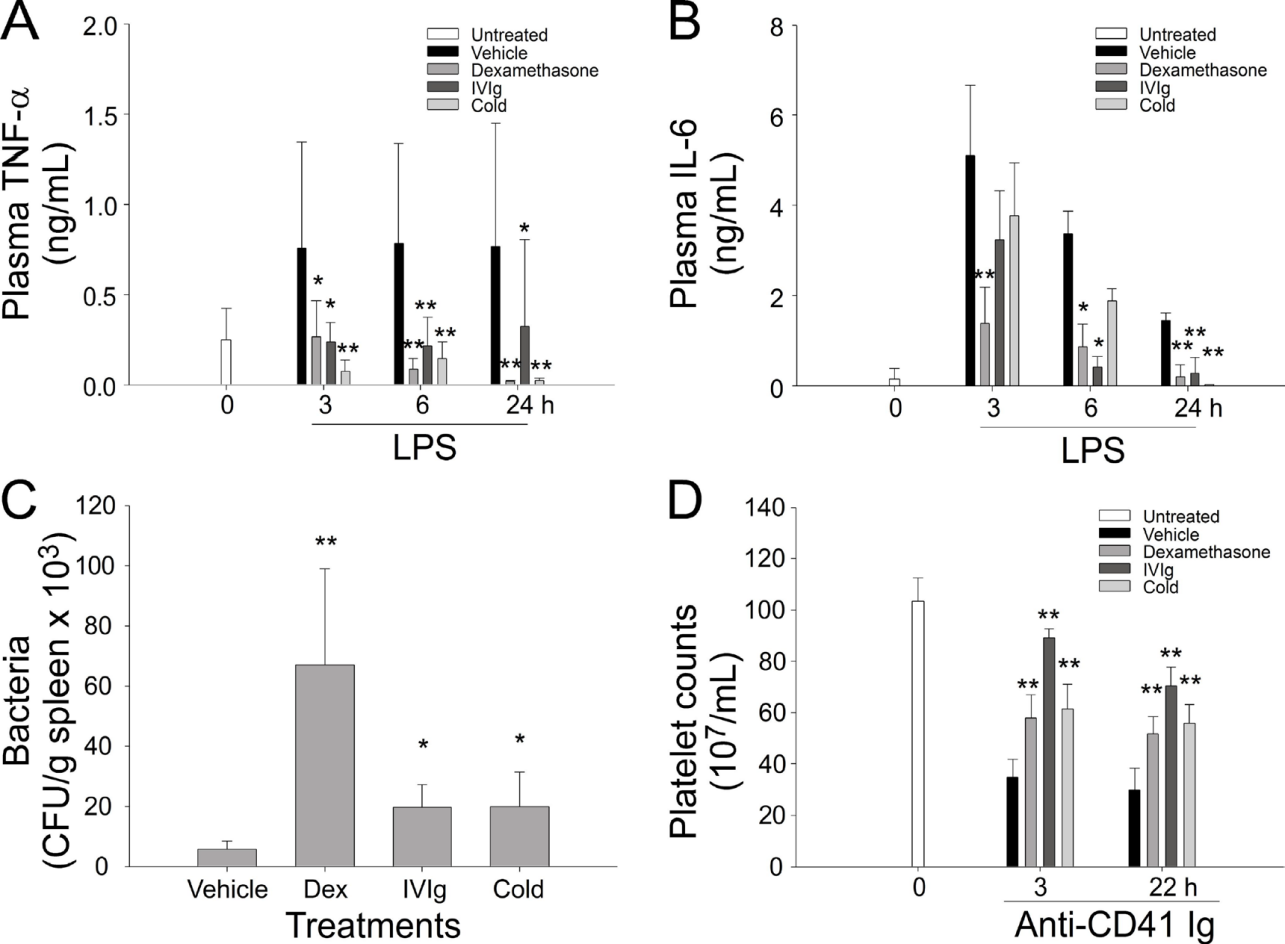
Cold exposure, dexamethasone, and intravenous immunoglobulin (IVIg) mediated anti-inflammatory effects in mice Cold exposure, dexamethasone, and IVIg resulted in the suppression of the lipopolysaccharide (LPS)-induced production of the plasma proinflammatory cytokines TNF-α (**A**) and IL-6 (**B**), bacterial clearance (**C**), and anti-CD41 Ig-mediated thrombocytopenia (**D**). ^*^P < 0.05 and ^**^P < 0.01 compared with respective vehicle control groups. *n* = 12 in 0-h groups (A, B, and D) and vehicle groups in C (six experiments with two mice per group); all other groups, *n* = 6 (three experiments with two mice per group).

The administration of a high dose of IVIg to increase circulating Ig levels is an effective treatment for autoimmune and inflammatory diseases, such as ITP and Kawasaki disease [11]. Because cold exposure increased plasma IgG levels (Figures 1F, 1G and 2B), we investigated whether cold exposure can lead to immunosuppression in mice. We found that cold exposure exerted a comparable immunosuppressive effect on the lipopolysaccharide (LPS)-induced production of the proinflammatory cytokines TNF-α and IL-6 compared with treatments with the well-recognized anti-inflammatory drugs dexamethasone and IVIg in mice (Figure 3A and 3B). Notably, treatment with dexamethasone, IVIg, and cold exposure also exerted an immunosuppressive effect on bacterial clearance in a sepsis mouse model (Figure 3C). Similarly, in the anti-CD41 Ig-induced ITP model, treatment with dexamethasone, IVIg, and cold exposure all markedly ameliorated thrombocytopenia in mice within 3 h (Figure 3D). These results suggest that cold exposure can elicit a rapid and robust anti-inflammatory response in mice.

### Cold exposure increased the plasma IgG level and induced IVIg-like immunosuppression in mice

**Figure 4:**
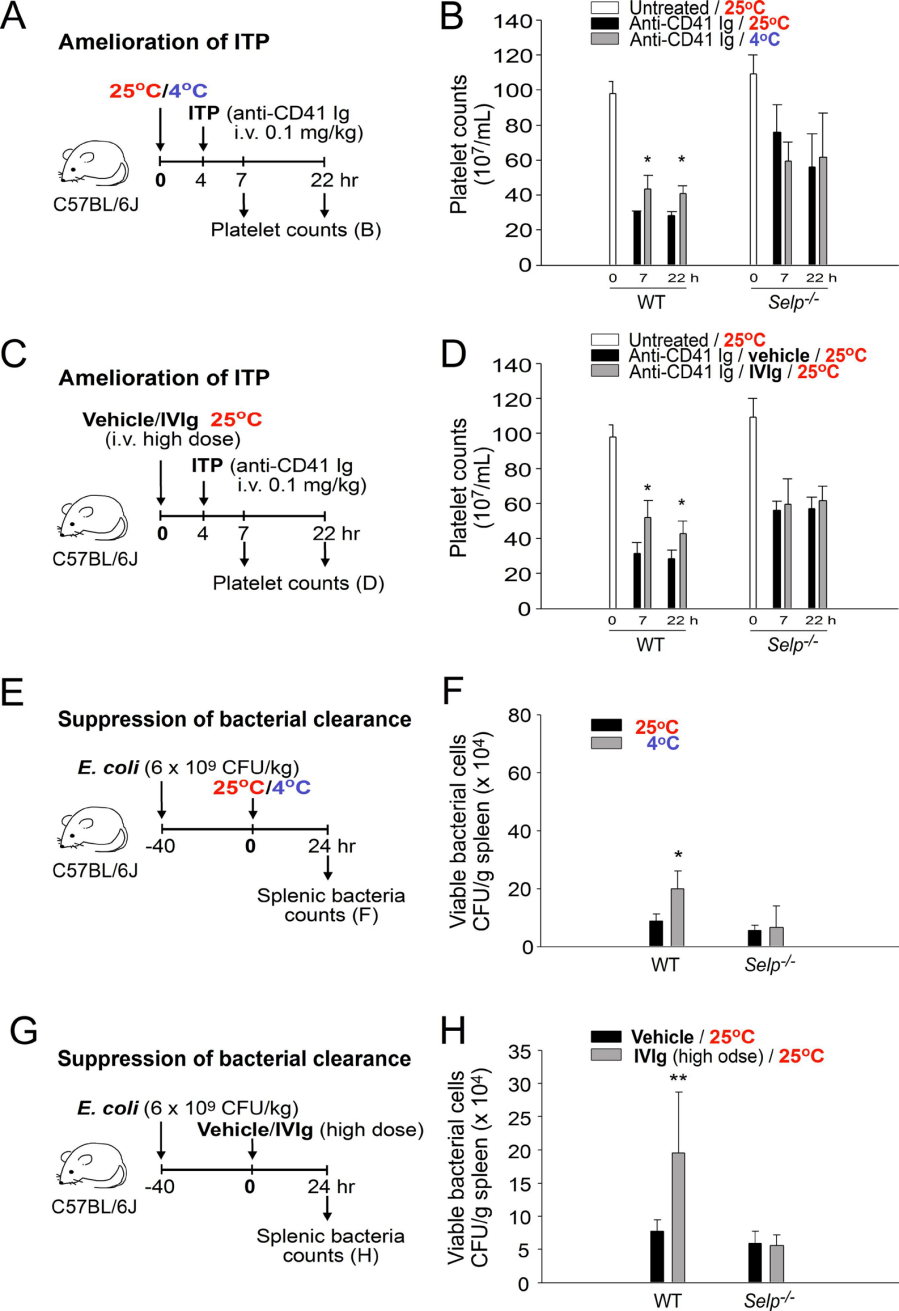
Treatment with cold exposure and intravenous immunoglobulin (IVIg) reduced immune thrombocytopenia (ITP) and bacterial clearance in mice Experiment outlines (**A**, **C**, **E**, and **G**). The platelet counts of the anti-CD41 immunoglobulin (Ig)-injected ITP mice [wild-type (WT) and *Selp^−/−^*] were analyzed 24 h before and 7 and 22 h after placement in a 4° C environment (A and B) or IVIg treatment (C and **D**; high dose, 1 g/kg); a group placed in a 25° C environment (**B**) and a vehicle-treated group (D) served as controls. The surviving bacteria (colony forming unit [CFU]) were quantified from the spleen tissues of mice (WT and *Selp^−/−^*) that were placed in 25° C and 4° C environments (E and **F**) or treated with a high dose of IVIg (1 g/kg; immunosuppressive) and a vehicle (G and H) for 24 h. All data in the figures are presented as the mean ± standard deviation (SD). The data are representative of three independent experiments with two replicates (*n* = 6). ^*^P < 0.05 and ^*^ *P* < 0.01 compared with the respective 25° C (B and F) and vehicle-treated (D and **H**) groups.

Because an increase in the plasma Ig level may lead to IVIg-like immunosuppression, gene knockout mice with a loss-of-function phenotype of IVIg-mediated immunosuppression should behave in a similar manner to that in cold-induced immunosuppression. For example, P-selectin is essential for transmitting IVIg-induced anti-inflammatory signals [21, 27]. Theoretically, P-selectin knockout (*Selp^−/−^*), but not wild-type, C57Bl/6J mice should show less immunosuppression under cold exposure. Therefore, we performed the ITP amelioration experiment, which is commonly used to verify the immunosuppressive property of IVIg, to compare the immunosuppressive effect of the IVIg treatment (1 g/kg body weight) [21] and cold exposure. As expected, cold exposure–induced ITP amelioration was observed in wild-type mice but not in P-selectin-deficient mice (Figure 4A, experiment outline, and Figure 4B); the rescue response was consistent with IVIg-mediated amelioration (Figure 4C and 4D).

IVIg can suppress antibacterial immunity [28, 29]. The bacterial clearance results of our study indicated that both IVIg treatment and cold exposure reduced bacterial clearance, and the inhibition patterns of both the groups are consistent with the results of the ITP amelioration analysis (Figure 4E; 4G, experimental outline; 4F; and 4H; the effect was observed in wild-type but not *Selp^−/−^* mice). These results suggest that the P-selectin pathway is essential in both cold exposure- and IVIg-mediated immunosuppression.

### Induction of circulating IgG but not of circulating cortisol and corticosterone is essential for cold-induced immunosuppression

**Figure 5:**
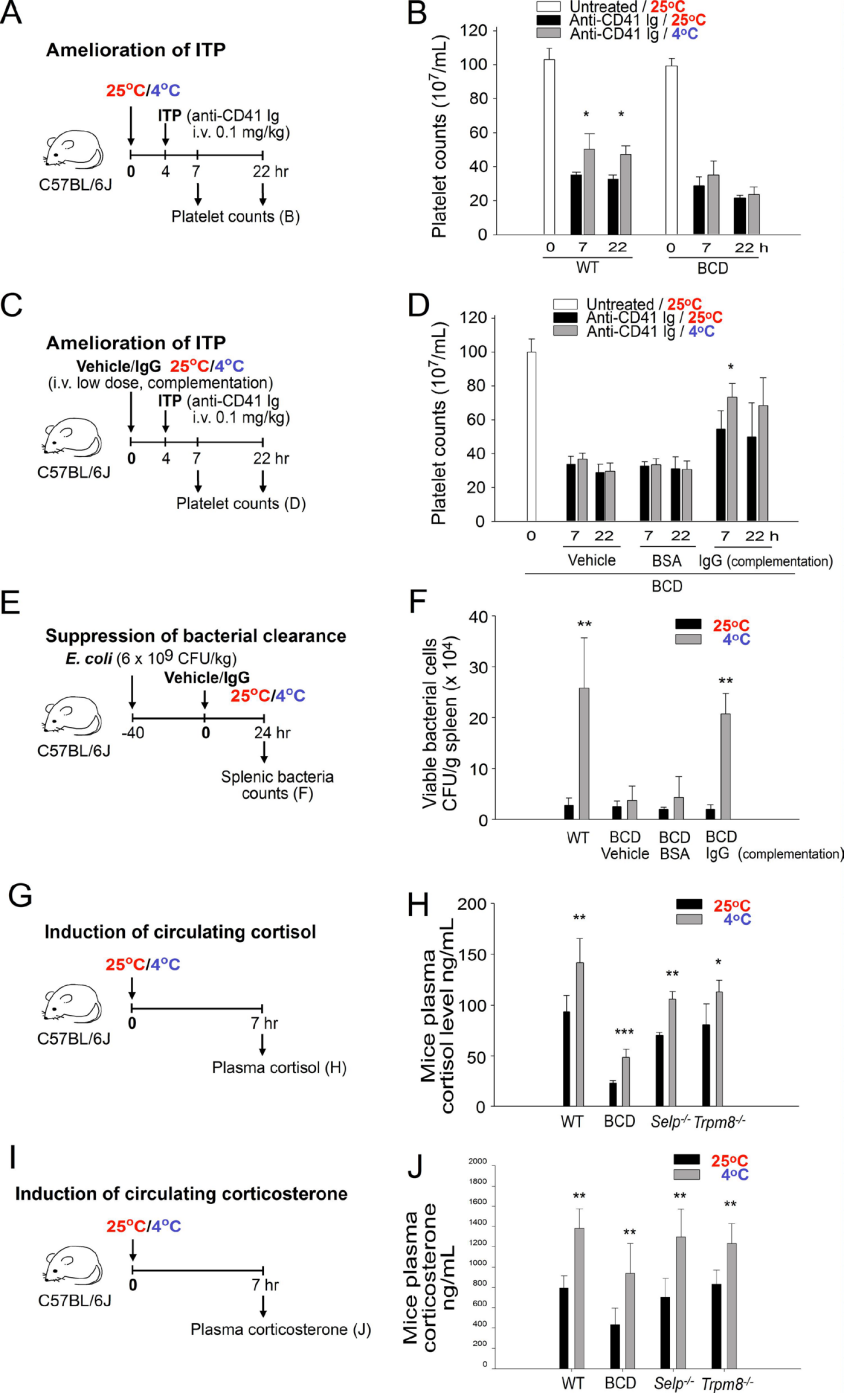
Induction of plasma immunoglobulin G (IgG) but not of cortisol and corticosterone is essential for cold exposure–induced immunosuppression Experiment outlines (**A**, **C**, **E**, **G**, and **I**) for cold-induced immune thrombocytopenia (ITP) amelioration (A–D), bacterial clearance (E and F), and cortisol and corticosterone (G–J) experiments. The platelet counts of the anti-CD41 Ig-injected ITP mice [wild-type (WT) and B cell-deficient (BCD; without Ig complementation)] were analyzed 0, 7, and 22 h after placement in a 4° C environment; a group placed in a 25° C environment served as controls (**B**). In 25 and 4° C environments, WT mice were compared with BCD mice with Ig complementation (IgG, 0.4 g/kg) of their ITP amelioration (**D**) and the levels of splenic surviving bacteria (**F**). The plasma cortisol (**H**) and corticosterone (**J**) levels of mice with or without cold exposure were compared among WT, BCD, *Selp^−/−^*, and *Trpm8^−/−^* groups. All data in the figures are presented as the mean ± standard deviation (SD) and are representative of three independent experiments with two mice per group (*n* = 6). ^*^ *P* < 0.05, ^**^ *P* < 0.01, and ^***^P < 0.001 compared with respective 25° C groups.

BCD mice (*Ighm^−/−^*) with no circulating Ig expression were subsequently used to determine whether Ig expression is essential for cold-induced immunosuppression. The results revealed that cold exposure–induced ITP amelioration was observed in wild-type but not in BCD mice, indicating that the expression of circulating Ig is essential (Figure 5A, experiment outline, and Figure 5B). To clarify whether in addition to the absence of Ig expression, BCD mice contain other defects that are critical for IVIg signal transmission, a complementation experiment involving the administration of an intravenous low-dose IgG injection (0.4 g/kg) was performed. The experimental results indicated that the complementary injections of IgG but not of a vehicle or control protein, bovine serum albumin, sufficiently restored cold-induced immunosuppression in BCD mice (Figure 5C, experiment outline, and Figure 5D; anti-CD41 Ig, 25° C vs. 4° C, 7-h groups). In agreement with the ITP analysis, cold exposure did not suppress bacterial clearance, unless BCD mice were administered complementary IgG injections (Figure 5E and 5F; BCD, Ig groups). These results collectively suggest that the expression of normal plasma IgG levels is critical for cold-induced immunosuppression.

Because the HPA axis-mediated induction of immunosuppressive steroids, such as cortisol, has been associated with cold exposure [8, 9], this study examined the plasma levels of the anti-inflammatory steroid hormones cortisol and corticosterone in wild-type and mutant mice under cold exposure. We found that cold exposure robustly increased plasma cortisol and corticosterone levels in wild-type, BCD, *Selp^−/−^*, and *Trpm8^−/−^* mice (Figure 5G–5J), whereas RAAS inhibitors could not suppress the increase (Supplementary Figure 3). These results suggest that the increase of cortisol and corticosterone levels is not an Ig-, P-selectin-, TRPM8-, and RAAS-dependent response. Because BCD (*Ighm^−/−^*)- and P-selectin (*Selp^−/−^*)-deficient mice displayed no ITP amelioration and no suppression of bacterial clearance following cold exposure (Figures 4 and 5A–5FA), these results suggest that the cold exposure-mediated increase of cortisol and corticosterone levels is not sufficient to induce immunosuppression.

### Cold exposure induces hypertension and increases plasma Ig levels through the TRPM8 pathway in mice

**Figure 6:**
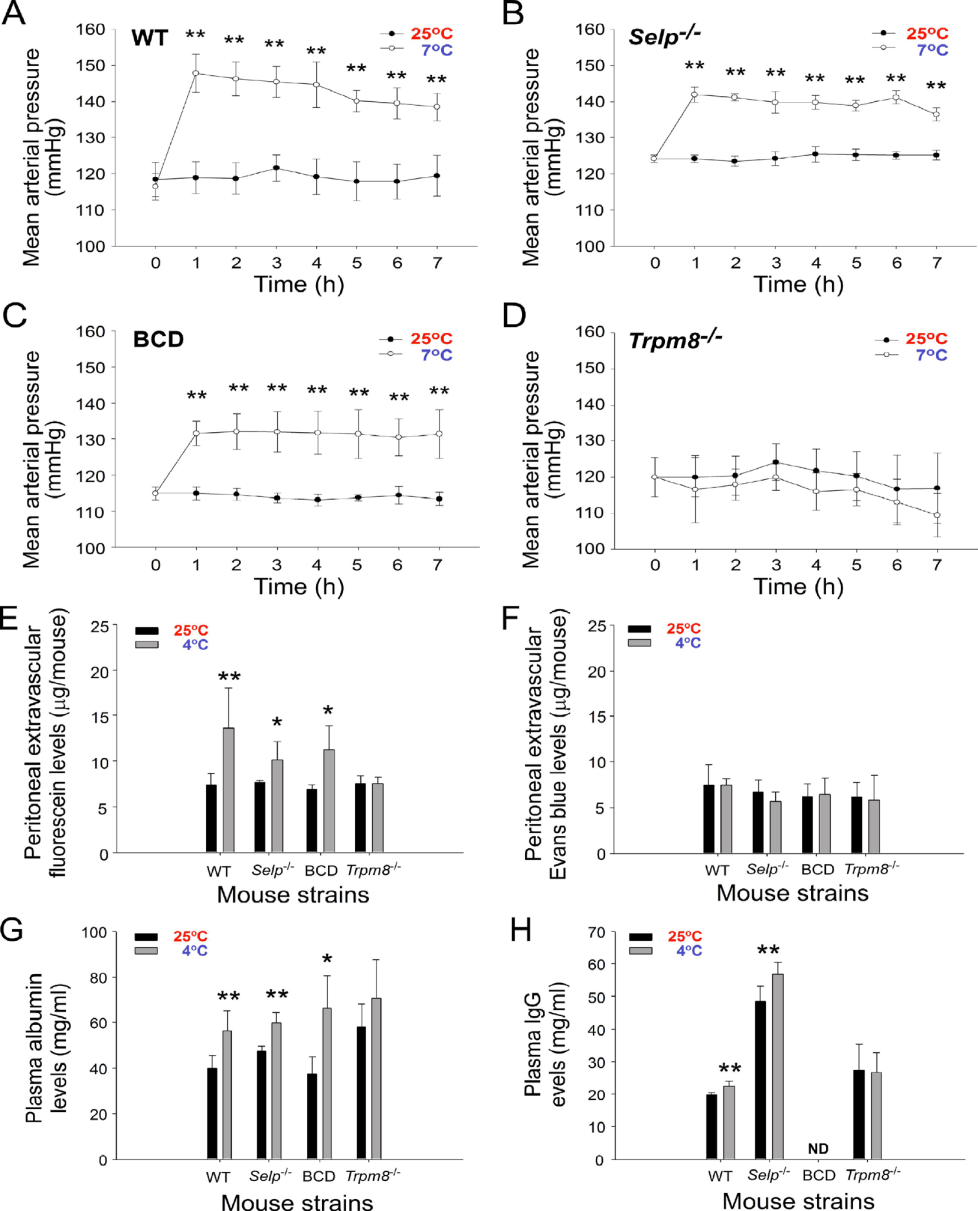
Cold-induced hypertension is associated with the elevation of small-molecule extravasation and increased plasma albumin and immunoglobulin G (IgG) levels The mean arterial pressure of wild-type (WT, adopted the data from Figure 1B) (**A**), *Selp*^−/−^ (**B**), B cell-deficient (BCD, *Ighm*^−/−^) (**C**), and *Trpm8*^−/−^ (**D**) mutant mice during cold exposure for up to 7 h is shown. The levels of the extravascular dyes fluorescein (**E**) and Evans blue in the peritoneum (**F**), and the levels of plasma albumin (**G**) and IgG (**H**) in mice with (4° C) or without (25° C) cold exposures are shown. Data are representative of three independent experiments with two mice per group (*n* = 6). ^*^P < 0.05 and ^**^P < 0.01 compared with respective earlier 4° C groups.

We investigated the involvement of the cold sensor TRPM8 because TRPM8 regulates the physiology in response to cold exposure [22, 30]. To investigate role of TRPM8 in the regulation of RAAS, we found that circulating renin activity and aldosterone levels were upregulated in wild-type, BCD and *Selp^−/−^* mice but not in *Trpm8^−/−^* mice during cold exposure (Supplementary Figures 4, 5). Consistently, gene expression of RAAS components, such as renin, AT1a, AT1b, AT2 and ACE, all markedly increased in wild-type but not in *Trpm8^−/−^* mice after cold exposure (Supplementary Figure 2). These results suggest that TRPM8 is an essential upstream regulator that triggers the hypertensive response through the RAAS. Consequently, we further analyzed the blood pressure, levels of the extravascular dyes fluorescein and EB in the peritoneum, and levels of albumin and IgG in the blood flow of wild-type and *Trpm8^−/−^* mutant mice following cold exposure. The results were consistent with our hypothesis that the mean arterial pressure and levels of the extravascular small molecule dye fluorescein and plasma albumin and IgG increase after cold exposure in wild-type and *Selp^−/−^* mice, except in *Trpm8^−/−^* mutant mice (Figure 6A–6DA, 25° C vs. 7° C and Figure 6E–6HE, 25° C vs. 4° C). BCD mice did not express Ig (Figure 6H); otherwise, the aforementioned phenotypes of BCD mice are similar to those of wild-type mice (Figure 6).

Compared with fluorescein, intravascular EB can bind to albumin (69,000 daltons) and form high-molecular-weight complexes in the blood stream [26]. Thus, the lack of peritoneal extravascular EB (Figure 6F, wild-type, *Selp^−/−^*, and BCD *Ighm^−/−^* mice, 25° C vs 4° C) indicated the retention of circulating albumin and larger molecules, such as IgG, in the mouse blood flow under cold exposure. These results suggested that cold exposure–induced hypertension is associated with an increase in plasma IgG levels and small-molecule extravasation, and TRPM8 is involved in all three responses (Figure 6). By contrast, given that P-selectin deficiency did not block the cold-induced elevation of circulating IgG level (Figure 6H) and that P-selectin knockout mice displayed a loss-of-function phenotype of cold-induced immunosuppression (Figure 4), P-selectin is likely a downstream pathway of TRPM8 and the RAAS, which is involved in transmitting high-circulating Ig-induced IVIg-like immunosuppressive signals as we have described previously [21].

### TRPM8 pathway is involved in cold exposure–induced immunosuppression

**Figure 7:**
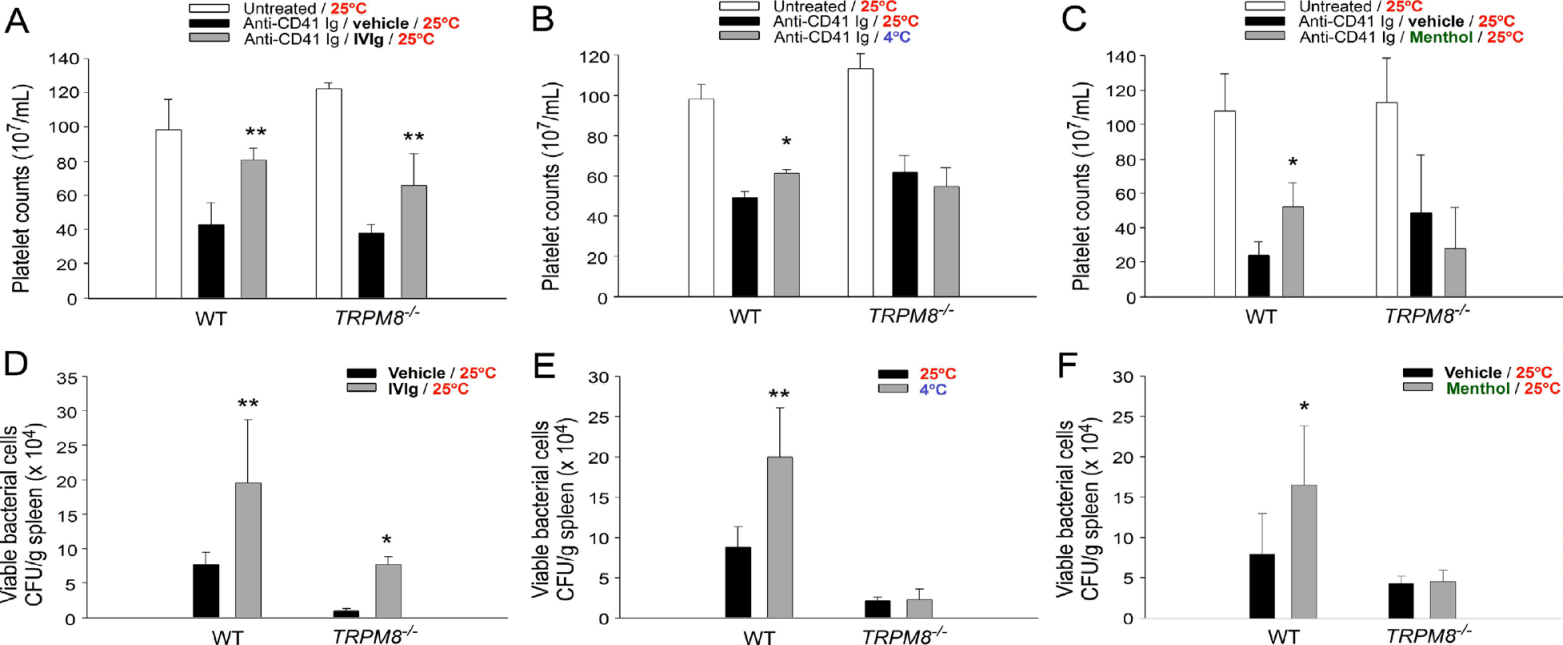
TRPM8 mediates cold-induced immunosuppression The differential responses of wild-type (WT) and *Trpm8^−/−^* mice to intravenous immunoglobulin (IVIg)-mediated ITP rescue (**A**) and reduced bacterial clearance (**D**) were compared with the responses to cold- (4° C) or menthol-induced (1 mg/kg) immune thrombocytopenia (ITP) amelioration (**B** and **C**) and suppressed bacterial clearance (**E**, **F**). The data are representative of three independent experiments with two mice per group (*n* = 6). ^*^P < 0.05 and ^**^P < 0.01 compared with the respective vehicle (A, C, D, and F), and 25° C (B and E) groups.

Because our results revealed that the cold-induced elevation of blood pressure and plasma protein levels (albumin and IgG) was not typically observed in *Trpm8^−/−^* mutants (Figure 6, WT vs. *Trpm8^−/−^*), TRPM8 knockout mice were further used to investigate the involvement of TRPM8 in cold-induced immunosuppression. IVIg treatments were functional to rescue ITP (Figure 7A), indicating that molecular and cellular machineries remain functional in *Trpm8^−/−^* mice to perform high plasma Ig level–induced immunosuppression. However, the cold exposure-mediated amelioration of ITP was nullified in *Trpm8^−/−^* mice (Figure 7B). Consistently, the IVIg-mediated suppression of bacterial clearance was functional (Figure 7D), whereas the cold-induced immunosuppression in *Trpm8^−/−^* mice was nullified (Figure 7E). Consistent with cold exposure, an injection of a considerable amount of menthol (1 mg/kg), a TRPM8 agonist, also suppressed ITP and bacterial clearance in wild-type mice but not in *Trpm8^−/−^* mice (Figure 7B and 7E vs. 7C and 7F, respectively). In agreement with the results of the cold exposure experiment (Supplementary Figures 4, 5), an increase in plasma aldosterone levels by menthol treatment was observed in only wild-type, BCD and *Selp^−/−^* mice but not in *Trpm8^−/−^* mice (Supplementary Figure 6). These results suggest that TRPM8 is critical in cold exposure–induced Ig-mediated immunosuppression.

### Cold exposure–induced immunosuppression is alleviated by antihypertensive treatment that reduces plasma Ig levels

**Figure 8:**
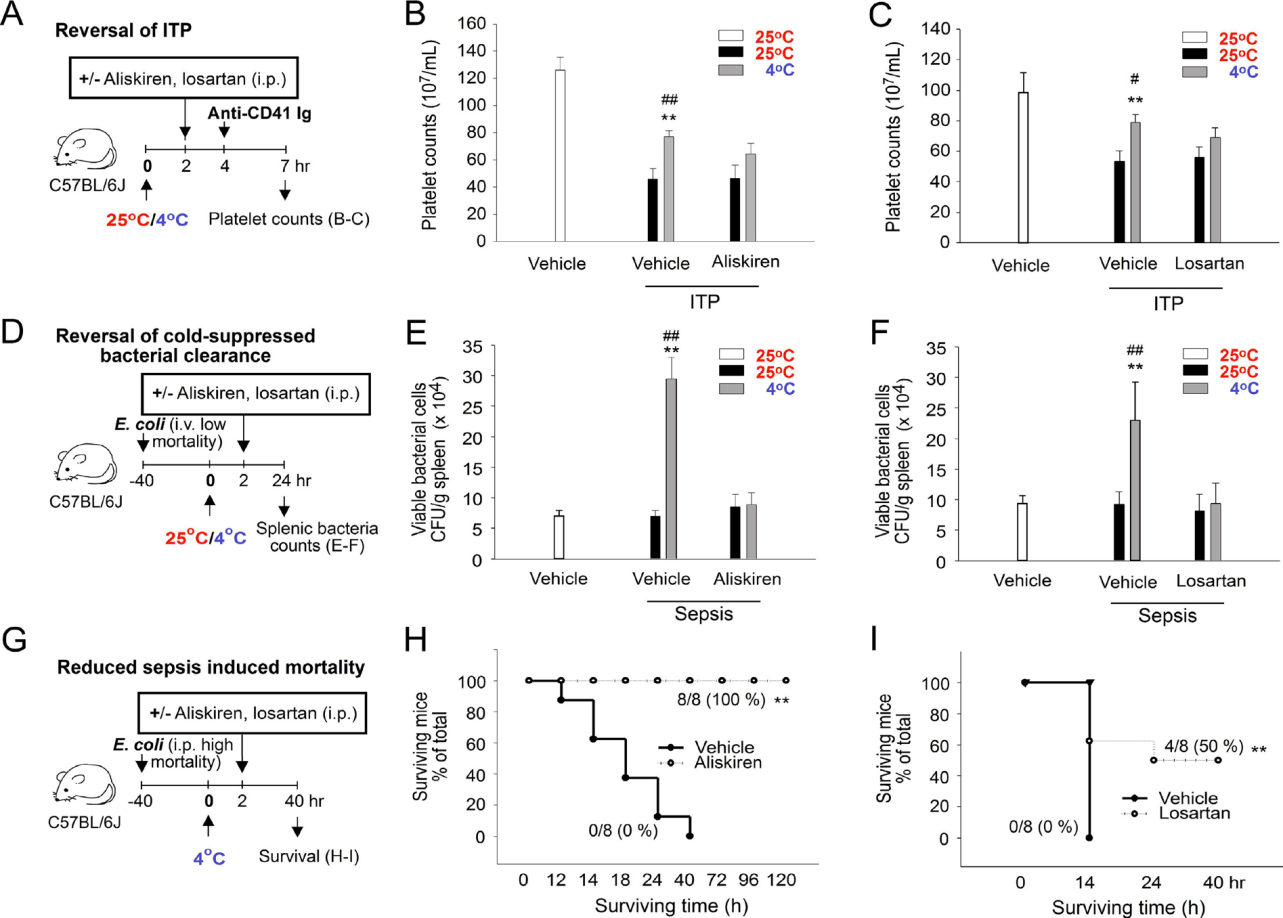
Rescue of cold-mediated immunosuppression by aliskiren and losartan treatments Experiment outlines (**A**, **D**, and **G**). The effects of aliskiren and losartan treatments on the cold-mediated amelioration of immune thrombocytopenia (ITP) (**B**–**C**), cold-suppressed bacterial clearance (**E**–**F**), and cold-induced mortality in septic mice (**H**–**I**) (Kaplan–Meier plot) were analyzed. The data are representative of three independent experiments with two (*n* = 6) (A–I) or two to three mice (*n* = 8) (H–I) per group. ^*^P < 0.05 and ^**^P < 0.01 compared with the respective vehicle (25° C) groups, ^#^*P* < 0.05 and ^##^*P* < 0.01 compared with the respective aliskiren and losartan 25° C groups (B, C, E, and F); ^**^P < 0.01 compared with the without rescue groups (H, I).

Transiently elevated blood pressure and the resultant Ig levels are likely the driving forces that increase plasma Ig levels and induce IVIg-like immunosuppression. We investigated whether this immunosuppressive effect can be overcome by antihypertensive interventions. Treatment with the antihypertensive drugs aliskiren and losartan significantly reversed the cold exposure-mediated elevation of plasma IgG levels (Figure 1F and 1G), indicating an alleviation of the IVIg-like response. In agreement with our hypothesis, both aliskiren and losartan treatments reversed cold-induced ITP amelioration (Figure 8A–8C) and suppressed bacterial clearance (Figure 8D–8F). In the mortality experiment, all mice survived the intraperitoneal injection of *Escherichia coli* at 25° C (*n* = 8), whereas the same treatment resulted in 100% mortality at 4° C within 14 h (Figure 8G–8I). Notably, both aliskiren and losartan treatments markedly reduced the cold exposure–induced mortality in septic mice (Figure 8H–8I, ^**^P < 0.01). In control groups, C57BL/6J mice survive at 4° C exposure for more than 2 weeks (Supplementary Figure 7). These results suggest that the antihypertensive treatment with both aliskiren and losartan is feasible for alleviating cold exposure–induced immunosuppression.

## DISCUSSION

**Figure 9:**
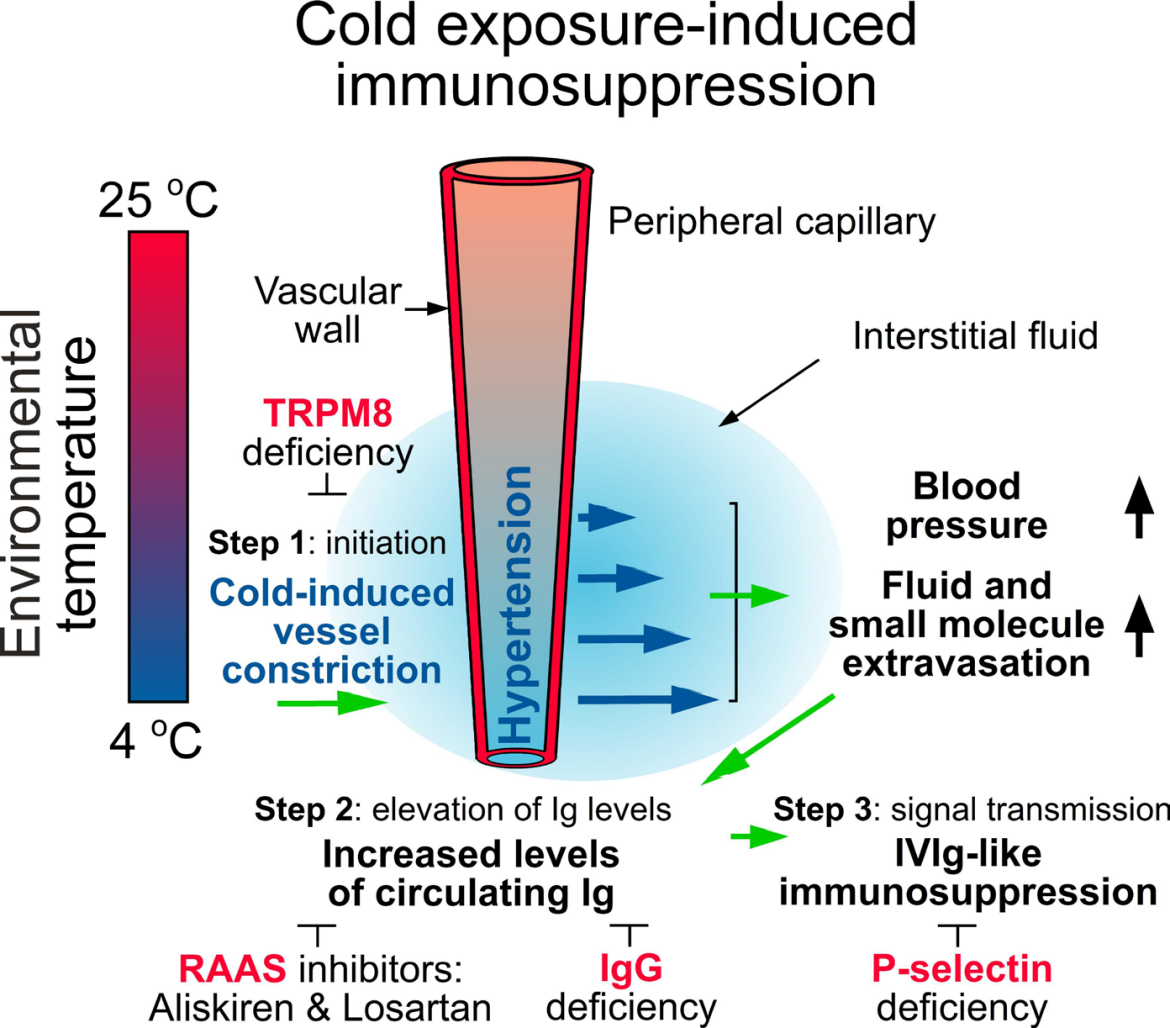
A hypothetical model To prevent heat loss, cold exposure leads to TRPM8-dependent RAAS-mediated hypertension and thus enhances fluid and small-molecule extravasation (step 1, TRPM8 dependent). This further increases the levels of plasma IgG (step 2, IgG dependent) and then elicits IVIg-like immunosuppression (step 3, P-selectin dependent). Antihypertensive treatment with the RAAS inhibitors aliskiren and losartan can ameliorate such progression. Green arrows: inductions. Large black arrows: increases. Small black arrows: indications and regulations. Blue arrows: fluid and small-molecule extravasation. ┴ Inhibition processes. Red words: four essential factors for cold-induced immunosuppression.

Global climate changes result in fluctuating and extreme weather patterns, such as a polar vortex, that affects public health worldwide [31, 32]. Cold exposure is associated with increased outbreaks of various infectious diseases [3–5]; however, the underlying mechanisms remain unclear. In addition, the immunosuppressive effects of IVIg are well known, and IVIg has been used for treating autoimmune or inflammatory diseases for decades [33]. However, whether a native IVIg-like response is present, which increases plasma Ig levels to suppress immune response in a native condition, remains elusive. In this study, we demonstrated the involvement of TRPM8-mediated elevation of plasma Ig levels in cold exposure-induced immunosuppression (Figure 9, hypothetical model).

Several studies have hypothesized that cold-induced immunosuppression is mediated through HPA axis responses, such as cortisol and corticosterone secretion [8, 9]. However, our data revealed that the examined mouse strains (e.g., TRPM8-, Ig-, and P-selectin-deficient mutants), which exhibited no obvious immunosuppression following cold exposure (Figures 4 and 5), did exhibit marked secretion of cortisol and corticosterone (Figure 5H, 5J), suggesting that the induction of cortisol and corticosterone alone is insufficient to induce cold exposure-mediated immunosuppression in our model.

According to the functional roles of the three essential factors TRPM8, Ig, and P-selectin, we can distinguish the progression of cold exposure–induced immunosuppression into three steps: step 1, the initiation of TRPM8-dependent RAAS-mediated hypertension; step 2, the elevation of circulating IgG levels; and step 3, the transmission of IVIg-like immunosuppressive signals (Figure 9). The involvement of RAAS in cold-induced hypertension is in agreement with previous reports [12]; for example, knockdown or knockout RAAS components angiotensinogen and angiotensin II receptors AT1a ameliorated cold-induced hypertension [24, 34]. TRPM8 is essential for the initiation of cold-induced RAAS-mediated hypertension, the extravasation of small molecules, and the elevation of the plasma Ig level in the step 1 (Figure 6 and Figure 9). IgG is the basis for the elevation of the circulating Ig level in the step 2 (Figure 5 and Figure 9). In the step 3, the elevated circulating IgG levels trigger a yet unidentified IgG level sensor in circulation and then transmit an immunosuppressive signal, similar to that in the IVIg treatment, in a P-selectin-dependent manner (Figure 4 and Figure 9).

Cold-induced immunosuppression is likely involved in seasonal infectious diseases contracted during the periods of cold weather [3–5, 7]. Safe and effective treatments for such cold exposure–induced adverse effects are urgently required. Because TRPM8 mediates cold-induced immunosuppressive effects, relevant antagonists may be candidates for alleviating the immunosuppression; however, the adverse effects of these reagents remain major concerns [35]. The antihypertensive drugs aliskiren and losartan are well-studied RAAS antagonists, which have been approved by the Food and Drug Administration of many nations including the United States. Because aliskiren and losartan can alleviate hypertension, these drugs may be useful for treating immunosuppression and sepsis-associated mortality (Figure 8) during the periods of cold weather.

In summary, the results of this study revealed that TRPM8 is a critical upstream regulator for eliciting cold exposure–induced RAAS-mediated hypertension. In addition, such cold exposure–induced hypertension can upregulate plasma Ig levels and further elicit Ig- and P-selectin-dependent immunosuppression. These data may provide novel perspectives regarding the management and prevention of cold exposure–induced immunosuppression and seasonal infections.

## MATERIALS AND METHODS

### IVIg and mice

IVIg was purchased from Bayer (Gamimune N, USA). C57BL/6J male mice (8–12 wk old), purchased from the National Laboratory Animal Center (Taipei, Taiwan), were used in experiments. C57BL/6J male mice (8–12 wk old) deficient in P-selectin (B6; 129S2-*Selp^tm1Hyn^*/J) [21, 36, 37], Ighm (BCD; B6.129S2-*Ighm^tm1Cgn^*/J), and TRPM8 (B6.129P2-*Trpm8^tm1Jul^*/J) were purchased from the Jackson Laboratory (Maine, USA). All animals were maintained in an specific-pathogen-free (SPF) facility in the Laboratory Animal Center of Tzu Chi University (Hualien, Taiwan). All research protocols for animal use were approved by and in accordance with the institutional guidelines (approval ID: 100054 and 103058).

### Measurement of the mouse blood pressure

For the blood pressure analysis, mice were anesthetized with intraperitoneal chloral hydrate (300 mg/kg; an anesthetic with limiting effects on blood pressure) and then placed in 25° C or 7° C (plastic plates on ice) environments. Because of the surgical operation, the mice became weak during the experimental course of blood pressure measurements; thus, a relatively milder cold exposure at 7° C was employed, as compared with 4° C that was used in other cold exposure experiments without surgical operation. During anesthesia, the femoral artery was cannulated and connected to a pressure transducer (MEMSCAP, Skoppum, Norway) to record arterial pressure on a polygraph recorder (eDAQ Pty Ltd., New South Wales, Australia). The operation was completed within 10 min, leaving a small wound (<0.5 cm^2^), and each mouse was placed on a cardboard. The mice were exposed to cold by placing a reusable cold pack (7° C; 3 M, Saint Paul, Minnesota, USA) under half of the cardboard under a mouse's body (head side), and a gauze pad was placed under the opposite side. Cold exposure was maintained for up to 7 h. To analyze the involvement of the RAAS, aliskiren (5 mg/kg; Novartis, Basel, Switzerland) and losartan (3 mg/kg; Merck & Co., Inc., Kenilworth, NJ, USA) were intraperitoneally injected 2 h after cold exposure.

### Measurements of renin activity

Mouse blood samples were collected from the retro-orbital venous plexus and mixed with an anticoagulant acid-citrate-dextrose solution (ACD; 38 mM citric acid, 75 mM sodium citrate, 100 mM dextrose) [21, 36, 37] in Eppendorf tubes. The plasma renin activity was measured using the Renin activity kit (Abcam, Cambridgeshire, UK). The fluorescent signal was measured using a microplate reader (Varioskan^TM^ Flash Multimode reader; Thermo Fisher Scientific, Waltham, MA, USA) at EX/EM = 540/590 nm.

### Measurements of gene expression of RAAS components

After 4° C cold exposure for 4 h, the total RNA of the mouse kidney and lung was isolated using a TRIzol™ reagent following the manufacturer's instructions (Thermo Fisher Scientific). For qRT-PCR, cDNA was first synthesized using the iScript cDNA Synthesis kit (Bio-Rad, Foster City, CA, USA), and then the qPCR reaction was performed using a Maxima SYBR Green/ROX qPCR Master Mix (Thermo Fisher Scientific) with primers (Supplementary Table 1) of target genes (renin, AT1a, AT1b, AT2 and ACE) and β-actin (an internal control). Results were analyzed using QuantStudio 5 qPCR and Thermo Flsher Cloud systems (Thermo Fisher Scientific).

### Measurements of peritoneal extravascular dyes

Fluorescein and EB (dissolved in 100 μL of normal saline; 1.6 g/kg; Sigma-Aldrich, St. Louis, MO, USA) were intravenously injected into the tail vein of wild-type and knockout mice as described [38, 39]. In time-course experiments, after placing these mice in 25 and 4° C environments for 1, 4, and 7 h, they were intraperitoneally injected with normal saline (2 mL/mouse). To analyze the involvement of the RAAS, aliskiren (5 mg/kg) and losartan (3 mg/kg) were intraperitoneally injected 2 h after cold exposure. The extravascular dye levels of peritoneal saline-containing exudates were then analyzed (4 and 7 h) using a multilabel plate reader (Perkin Elmer Life and Analytical Sciences, Boston, MA, USA) based on standard curves obtained from the serially diluted samples of fluorescein and EB.

### Measurements of aldosterone, cortisol, corticosterone, and plasma proteins

The plasma levels of cortisol, corticosterone, TNF-α, IL-6, and proteins (albumin, fibrinogen, and IgG) in the mice placed in 25 or 4° C environments for 4 h were measured using ELISA (aldosterone, cortisol, and corticosterone kits: Cayman Chemical, Ann Arbor, MI, USA; TNF-α and IL-6 kits: Biolegend, San Diego, CA, USA; antimouse albumin Ig: Bethyl Laboratories, Montgomery, TX, USA; antimouse IgG: Jackson Immunoresearch, West Grove, PA, USA; and antifibrinogen Ig: Abcam, Cambridge, MA, USA).

### Induction and rescue of inflammation and ITP

Bacterial lipopolysaccharide (LPS) is a typical positive-control agent for inducing inflammation [40–43]. LPS and anti-CD41 Ig were used to investigate the immunosuppressive property of cold exposure versus the well-known anti-inflammatory drugs dexamethasone and IVIg. LPS (80 μg/kg; *E. coli*; Sigma-Aldrich) was intravenously injected just before (0 h) placing these mice into a 4° C cold room to induce the production of the proinflammatory cytokines TNF-α and IL-6. Experimental ITP was induced as described previously [21, 44]. The mice were intravenously injected with 0.1 mg/kg (body weight) of an antiplatelet monoclonal antibody (rat antimouse integrin α_IIb_/CD41 Ig, clone MWReg30, BD PharMingen) to induce ITP. To analyze the platelet count, whole blood samples (100–120 μL) of the mice were collected from the retro-orbital venous plexus and mixed with an anticoagulant, a citrate dextrose solution (38 mM citric acid, 75 mM sodium citrate, and 100 mM dextrose), in Eppendorf tubes. Subsequently, platelet counts were measured using a hematology analyzer (KX-21N, Sysmex) at various time intervals. To investigate the ameliorative effects of dexamethasone, IVIg, menthol, and cold exposure on LPS-induced inflammation and anti-CD41 Ig-induced ITP, the mice were intravenously treated with dexamethasone (2 mg/kg; vehicle: DMSO dissolved in saline; four injections at 72, 48, 24, and 0 h), intravenously treated with IVIg (high dose, 2 g/kg; vehicle: saline; injection at 0 h), subcutaneously treated with menthol (1.6 mg/kg; Sigma-Aldrich; vehicle: corn oil, 250 mL/mouse; injections at 4 h), or placed in 4° C environments, respectively, before ITP induction (anti-CD41 Ig MWReg30 treatments). The antihypertensive drugs aliskiren (5 mg/kg; vehicle: saline) and losartan (3 mg/kg; vehicle: saline) were also used to overcome cold-induced immunosuppression (ITP amelioration). Both reagents were intraperitoneally injected 2 h before anti-CD41 Ig administration.

### Bacterial clearance and sepsis-induced mortality under cold exposure

The bacterium *E. coli* was cultured using standard methods [45–47]. To analyze the immunosuppressive effects of the treatments of IVIg, menthol, and cold exposure on bacterial clearance, the experimental mice were challenged by intravenously administering bacteria (*E. coli*, BL21, 6 × 10^9^ CFU/kg; no mortality within 24 h at 4° C); a 40-h circulating equilibrium at a 25° C environment was required before cold exposure (control groups: 25° C exposure) or intraperitoneal menthol treatment (1.6 mg/kg; vehicle: corn oil). One day after treatment with IVIg, menthol, or cold exposure, the spleens of the euthanized mice were collected and weighed. After homogenization (homogenizer, BioSpec Products, Racine, WI, USA) in PBS, surviving bacteria (colony forming units [CFUs]) in the spleen tissues were quantified using the standard plating method. Aliskiren (5 mg/kg) and losartan (3 mg/kg) treatments were used to rescue cold-induced immunosuppression; both reagents were intraperitoneally injected 2 h after cold exposure. To analyze the effects of cold exposure on sepsis-induced mortality, the mice were challenged by intraperitoneally administering bacteria (*E. coli*, BL21, 6 × 10^9^ CFU/kg; 100% mortality within 14 h at 4° C, no mortality at 25° C); a 40-h circulating equilibrium was required before placing the mice in 25 or 4° C environments.

### Statistical analysis

The means, standard deviation (SD), and statistics of the quantifiable data were calculated using Microsoft Office Excel 2003, SigmaPlot 10, and SPSS 17, respectively. The significance of the data was examined using one-way ANOVA, followed by the post hoc Bonferroni-corrected *t* test. A probability of type 1 error (α = 0.05) was recognized as the threshold for statistical significance.

## SUPPLEMENTARY MATERIALS FIGURES AND TABLE


