# Supplementary material for: TRPM8 and RAAS-mediated hypertension is critical for cold-induced immunosuppression in mice
Source: Oncotarget. 2018 Jan 30;9(16):12781–95. doi: 10.18632/oncotarget.24356 (PMC5849173; doi:10.18632/oncotarget.24356)
Supplement: Supplementary file 1 [file oncotarget-09-12781-s001.pdf]

TRPM8 and RAAS-mediated hypertension is critical for cold-induced immunosuppression in mice

SUPPLEMENTARY MATERIALS

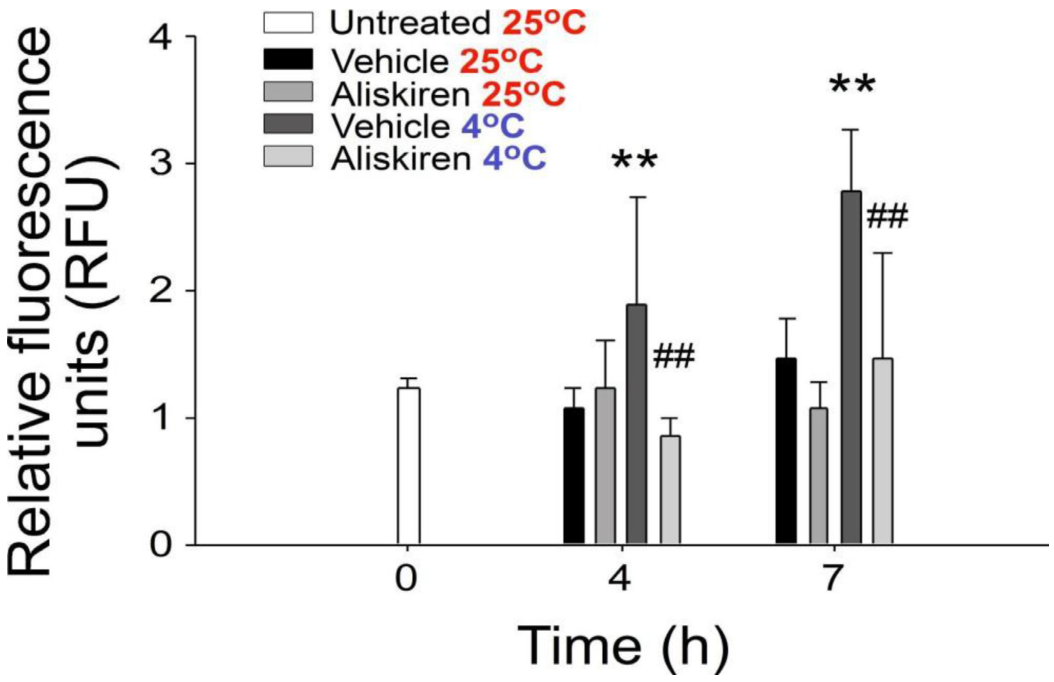

Supplementary Figure 1: The renin activity. Treatment with the antihypertensive drug aliskiren suppressed cold exposure–induced increase of circulating renin activity in the plasma of wild type mouse. Data in the figure are presented as the mean ± standard deviation (SD) and are representative of three independent experiments with two mice per group (n = 6). $^{**}P < 0.01$ compared with the respective vehicle 25°C groups; and $^{\#\#}P < 0.01$ compared with the respective vehicle 4°C groups.

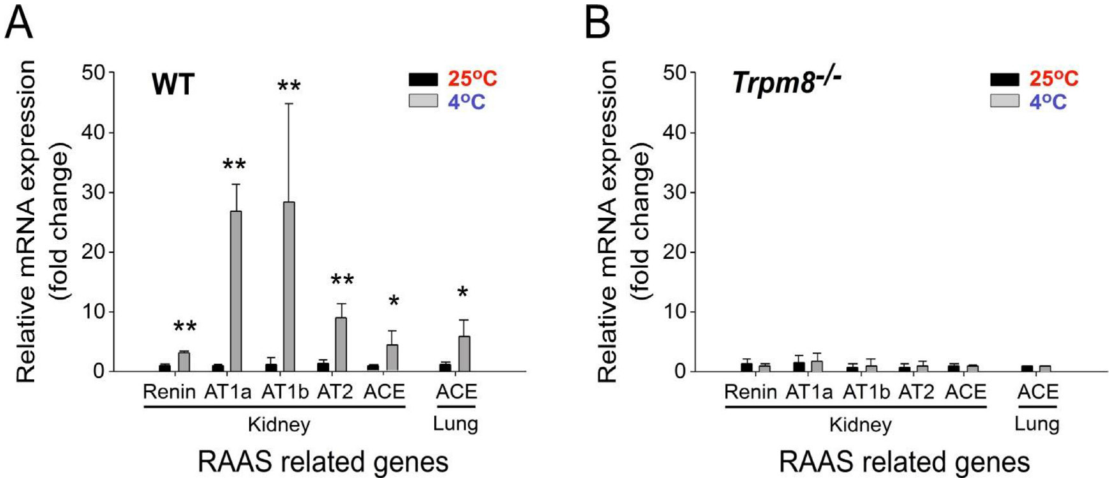

Supplementary Figure 2: Induction of RAAS-component gene expression after cold exposure in mice. Quantitative reverse transcription-polymerase chain reaction (qRT-PCR) analysis revealed that RAAS component genes, such as renin, angiotensin II receptors AT1a, AT1b, AT2 (primarily expressed in the kidney) and angiotensin converting enzyme (ACE; expressed in both kidney and lung) [1,2], all considerably enhanced after 4 h, 4°C cold exposure in wild type (WT) (**A**) but not in Trpm8 knockout (*Trpm8*$^{-/-}$) (**B**) mice. All results are normalized with corresponding actin mRNA expression. Data are presented as the mean ± standard deviation (SD) and are representative of two independent experiments with two and three mice per group (n = 6 in AT1a, AT1b groups, n = 4 in renin, AT2, ACE groups; each sample with triplicated analysis and counted as n = 1). $^{*}P < 0.05$, $^{**}P < 0.01$ compared with the respective 25°C groups.

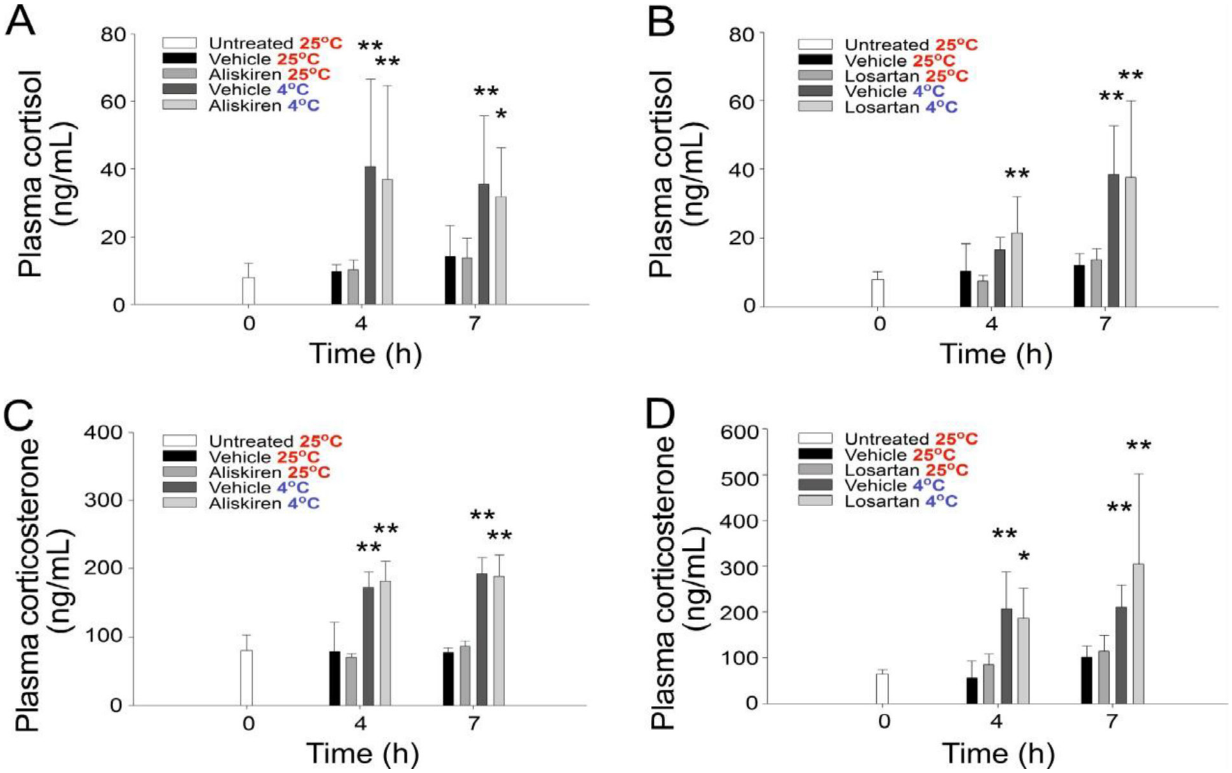

Supplementary Figure 3: Plasma cortisol and corticosterone levels in mice. Treatment with the antihypertensive drugs aliskiren and losartan did not suppress the cold exposure–induced increase in plasma cortisol and corticosterone levels. Cold exposure increased the levels of cortisol (**A**, **B**) and corticosterone (**C**, **D**) with or without aliskiren (A, C) and losartan (B, D) treatments. All data in the figures are presented as the mean ± standard deviation (SD) and are representative of three independent experiments with two mice per group (n = 6). $^{*}P < 0.05$ and $^{**}P < 0.01$ compared with the respective 25°C groups.

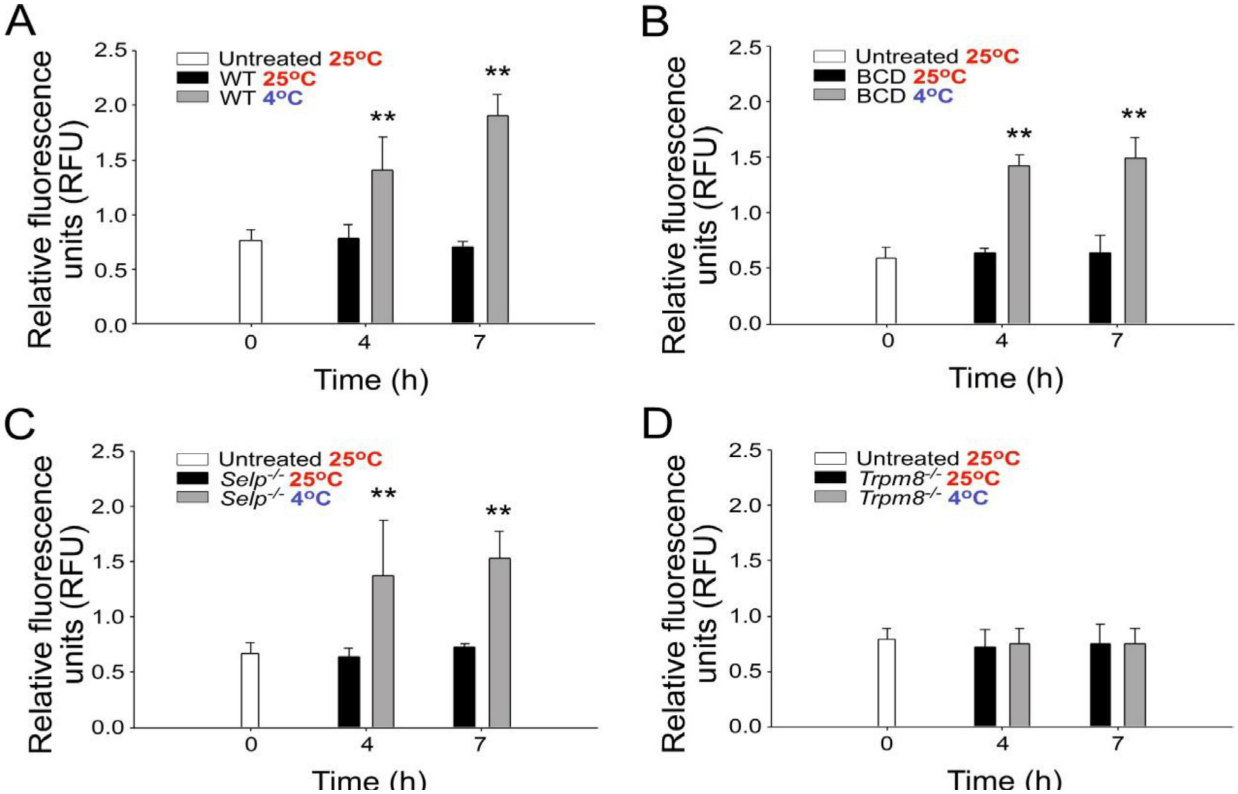

Supplementary Figure 4: The circulating renin activities of wild type and mutant mice. Differential behaviors after the induction of cold exposure-mediated increase of circulating renin activity in wild-type (WT) versus mutant mice. Cold exposure increased aldosterone levels in WT (**A**), B cell-deficient (BCD; **B**), P-selectin null *Selp*$^{-/-}$; (**C**), and TRPM8 null *Trpm8*$^{-/-}$; (**D**) mice. All data in the figures are presented as the mean ± standard deviation (SD) and are representative of two independent experiments with two or three mice per groups (A, B n = 10 in 0 h group, n = 5 in 4 h and 7 h groups; C, D n = 8 in 0 h group, n = 4 in 4 h and 7 h groups). $^{**}P < 0.01$ compared with the respective 25°C groups.

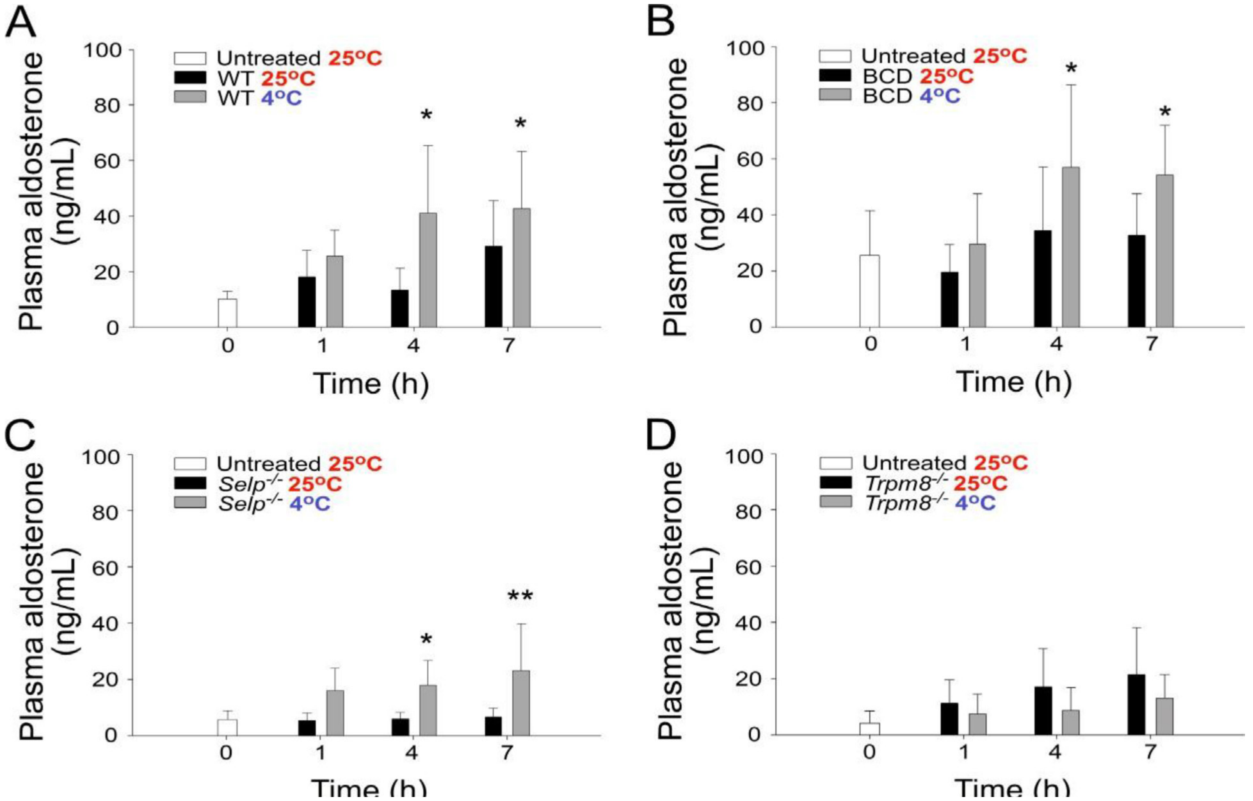

Supplementary Figure 5: Plasma aldosterone levels of wild type and mutant mice after cold exposure. Differential behaviors after the induction of cold exposure-mediated increase of aldosterone levels in wild-type (WT) versus mutant mice. Cold exposure increased aldosterone levels in WT (**A**), B cell-deficient (BCD; **B**), *Selp*$^{-/-}$ (**C**), and *Trpm8*$^{-/-}$ (**D**) mice. All data in the figures are presented as the mean ± standard deviation (SD) and are representative of three independent experiments with two mice per groups ($n = 6$). $^{*}P < 0.05$ and $^{**}P < 0.01$ compared with the respective 25°C groups.

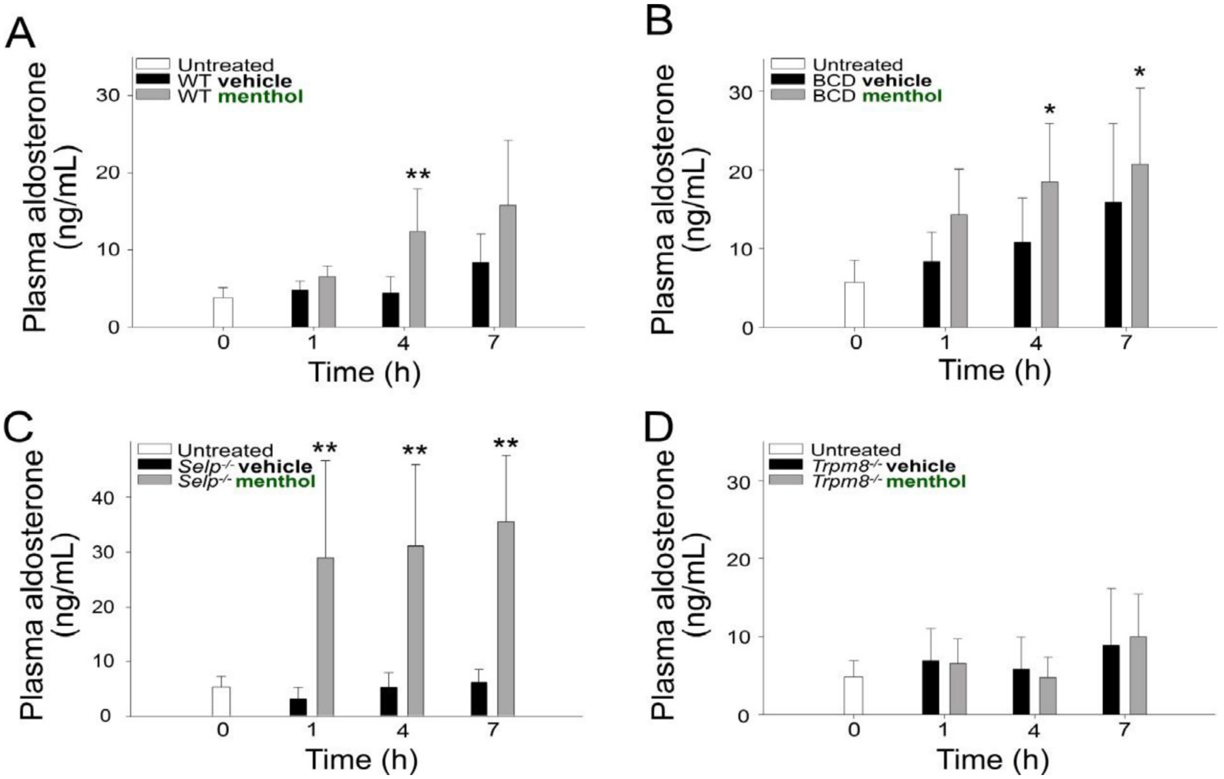

Supplementary Figure 6: Plasma aldosterone levels of wild type and mutant mice after menthol treatments. Differential behaviors after the induction of menthol-mediated increase in the aldosterone level in wild-type (WT) versus mutant mice. Menthol increased aldosterone levels in WT (**A**), B cell-deficient (BCD; **B**), *Selp*$^{-/-}$ (**C**), and *Trpm8*$^{-/-}$ (**D**) mice. All data in the figures are presented as the mean ± standard deviation (SD) and are representative of three independent experiments with two mice per group (n = 6). $^{*}P < 0.05$ and $^{**}P < 0.01$ compared with the respective 25°C groups.

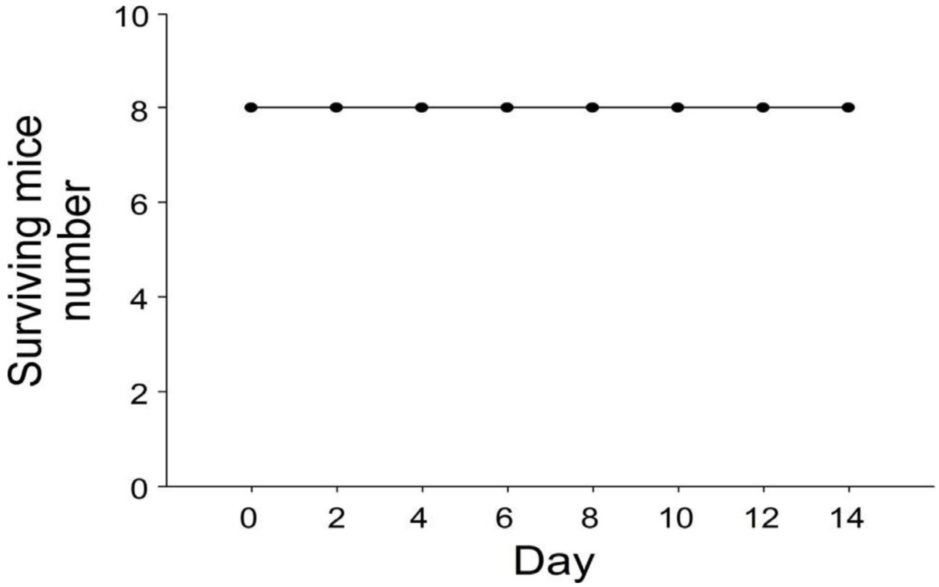

Supplementary Figure 7: Mortality of mice under cold exposure. No mortality occurred in wild-type C57BL/6J mice placed in the 4° C environment for two weeks (n = 8).

Supplementary Table 1: Primers used in the qRT-PCR analysis

RAAS component	Primers	Fragment size (bp)
Renin	5′-atctttgacacgggttcagc-3′	207
	5′-cacagtgattccacccacag-3′	
AT1a	5′-ggaaacagcttggtggtgat-3′	234
	5′-gagacacgtgagcaggaaca-3′	
AT1b	5′-caacttccagccttttcagg-3′	220
	5′-ccctcccccaaatcaatagt-3′	
AT2	5′-cccaggtccacagaactcat-3′	188
	5′-cagcatcaatccaggaaggt-3′	
ACE	5′-cagtgtctacccccaagcat-3′	165
	5′-ttccatcaaagaccctccag-3′	
β-actin	5′-atctggcaccacaccttcta-3′	136
	5′-gggtgttgaaggtctcaaac-3′	

REFERENCES

1. Huang J, Yamashiro Y, Papke CL, Ikeda Y, Lin Y, Patel M, Inagami T, Le VP, Wagenseil JE, Yanagisawa H. Angiotensin-converting enzyme-induced activation of local angiotensin signaling is required for ascending aortic aneurysms in fibulin-4-deficient mice. Sci Transl Med. 2013; 5:183ra158, 181–111.
2. Reinhold SW, Kruger B, Barner C, Zoicas F, Kammerl MC, Hoffmann U, Bergler T, Banas B, Kramer BK.. Nephron-specific expression of components of the renin-angiotensin-aldosterone system in the mouse kidney. J Renin Angiotensin Aldosterone Syst. 2012; 13:46–55.
